# Observations in Practice, Surgery, Gynecology, and Especially Obstetrics

**Published:** 1878-02

**Authors:** George B. Walker

**Affiliations:** Professor of Obstetrics in the Medical College of Evansville


### OBSERVATIONS IN PRACTICE, SURGERY, GYNECOLOGY, AND ESPECIALLY OBSTETRICS.

By George B. Walker, M. D.,

PROFESSOR OF OBSTETRICS IN THE MEDICAL COLLEGE OF EVANSVILLE.

*(Read before the Indiana, Illinois and Kentucky Tri-State Medical Society, in Evansville, Oct.* 17, 1877.)

The professional observations of the writer of this article extend over a period of forty-six years—five years in Cincinnati, and the remainder in Evansville. The diseases met with were those common to the two sections, and, to avoid prolixity, I will confine myself chiefly to cases belonging to surgery, gynecology, and especially obstetrics. Every practitioner of medicine is necessarily familiar with malarial and gastric fevers, erysipelas, dysentery, cholera, cholera infantum, and the vast variety of endemic, epidemic and contagious diseases of the country, including the exanthemata; and though familiarity can never destroy the interest felt in them, a particular description of them, including history, pathology and treatment, might be made to fill volumes, and would prove too extensive a subject to embody in a report like the present.

At the meeting of the Tri-State Society, held in Vincennes two years ago, the writer was appointed to make a report on anæsthetics, and also on observations and experience in practice, and to avoid consuming too much time, and since on reflection I concluded that I could not state anything of importance, new, or even as valuable as information already before the profession, especially the valuable and exhaustive contribution of Dr. Simpson, I have thought best to embody what I have to say on this important subject in the present essay. Simpson and others, who have taken pains to investigate anæsthetics thoroughly, have described quite a number of articles efficient to destroy the feeling of pain, and therefore I can do no better service than to refer to their works for information in detail. Let it suffice that opium, alcohol, ether, chloral, nitrous oxide gas and chloroform, constitute nearly everything desirable as anæsthetics in medical and surgical practice. Whilst many of our New England brethren prefer ether before chloroform, in old England, under the intelligent leadership of such men as Simpson, chloroform takes precedence. It is urged in favor of ether, that its inhalation is less dangerous to life, and in favor of chloroform, that it is more pleasant to the taste, and more speedy and reliable in securing the object desired. It is possibly true, that the greater rapidity and certainty of action of the latter article, are the qualities that make its use comparatively more hazardous. Anæsthesia, properly speaking, is, to a limited extent, a suspension of the vital functions, and, so far as this suspension extends, is the approach to death. Anæsthetics appear to paralyze the phenomena of life, in something like regular order: 1. By loss of sensation and voluntary motion. 2. By suspension of the heart's action. 3. By suspending the respiratory functions. Their utility depends upon their action being first expended on the organs of animal life, and their use may be considered safe so long as their administration is not carried beyond this point. As long as the circulation and respiration are not materially disturbed, the danger is at its minimum. It would not seem to be an unreasonable inference that in all efficient anæsthetics, the good and bad qualities are necessarily conjoined. If ether were equally prompt with chloroform in suspending sensation, its dangerous qualities would be similar. To avoid dangerous results, all anæsthetics must be used with caution, the circulation and respiration must be carefully watched—suspension of either would necessitate the immediate withdrawal of the anæsthetic, and the application of the means of restoration. Nitrous oxide gas is relatively more safe, in consequence of the shorter duration of its specific effects, but for this reason, it is not well adapted to the more tedious operations of surgery; for operations requiring but little time, like the operations of the dentist, it should generally have the preference; for longer infliction of pain, perhaps there is no remedy equal to chloroform inhalation. It seems to be a prevalent belief, that in certain morbid conditions of the heart and respiratory organs, anæsthetics are specially unsafe, and if used in such cases, the strictest precautions ought to be observed, by watching the functions of the vital organs. If a portion of the texture of the lungs should have been destroyed, or seriously damaged by disease, so as barely to suffice for the necessary aerification of the blood, it is very plain that to inhale gases devoid of oxygen, must, to a greater or less extent, suffocate the patient. It is but a reasonable conclusion, therefore, that such lungs would disqualify the patient from anæsthesia through inhalation.

From a fair and philosophical view of the advantages and dangers of anæsthetics, as administered by the lungs, to repudiate them entirely would be to inaugurate a retrogade movement of the profession. Beyond question, in surgical cases, many lives are saved through their use and few destroyed, whilst the benefit of painless operations is beyond comparison. The relaxation, the entire freedom from resistance or complaint, and the unconsciousness of pain during a serious operation, are among the benefits of anæsthesia. The relaxation following their use especially favoring obstetrical manœuvres, is of incalculable benefit, as every practioner of experience well knows. In delicate operations on timid persons and children, for instance on the eye, the advantage of securing perfect quietness is frequently more important than skill itself. The danger arising from shock, common in all serious operations, is almost entirely avoided by anæsthesia, and, in short, in the present enlightened state of surgical science, the operator who would fail to avail himself of this resource would be inexcusably culpable. To deny its use would frequently subject the patient to a painful apprehension of suffering, and a harrassing mental excitement, equal in degree to the pains inflicted by the surgeon, and ten-fold more dangerous to life than anæsthesia. When used to mitigate the pains of labor, as must occasionally be consented to by every practitioner, even to gratify the fancy of the patient, if for no other reason, it will generally be advisable to withhold its use to a late period, say when the perineal tumor is forming, and even then at the termination of each uterine contraction, otherwise its operation might be excessive and continued through an unreasonable period. With such precautions, the pains may be moderated without being entirely destroyed, the patient's mind will be at ease, and the labor completed with perfect safety and mitigated suffering. Its excessive use in such cases must cause the blood of the patient to be highly impregnated with the drug, and even the infant's blood also, as is manifested occasionally by its odor on dividing the cord. In obstetrical, as in other surgical operations, the anæsthetic must be more liberally applied, inasmuch as the insensibility should be more profound than in natural delivery. That the remedy has some effect on the uterine contractions to modify their strength in many instances, there can scarcely be a doubt. Generally it weakens the action, making the pains less frequent and less strong, the labor being retarded, not otherwise suspended. In some cases the contractions continue without change by increase or diminution, whilst in others again they are perceptibly increased. It is not unreasonable to suppose that perfect calmness of mind and freedom from excitement of the system generally, such as may be secured by anæsthesia, would favor a more healthful supply of nervous power to organs engaged in labor, and thus give increased strength for the accomplishment of the function. This would especially be the case in irritable women and those commonly denominated nervous. Finally, it is perfectly reasonable to assume that anæsthesia places in the hands of the physician and surgeon one of the most valuable resources for the mitigation of human suffering, and in view of its great advantages, a comparatively safe one.

*Ascites.*—1 *Case.*

Dropsy being a common disease, and the form of ascites being also common, I forbear entering upon a full account of the malady, but have deemed a single case of sufficient interest, to make a general report of it.

Mrs. S——, aged about 50 years, mother of three or more children, had suffered for several years from visceral disease, now become chronic, of the organs of the abdomen, which culminated in ascites. The patient refusing the necessary treatment, the accumulation in the peritoneal sac increased, until the pain caused thereby could no longer be endured, and paracentesis was at length submitted to, when, 7 gallons of serous fluid came away. In about 5 months the operation was repeated, and the same large quantity drawn off. After this time until the present, September 23d, 1877, the intervals between operations have grown shorter by degrees, until now, the tapping is required about once a month, the quantity drawn off each time, never being less than 5 gallons, and has averaged no less than 6 gallons of fluid. The patient has been operated on 34 times, to wit: First on March 7, 1872; the last, September 23, 1877. The whole amount drawn off, therefore, is no less than 204 gallons. At present writing, the patient shows little sign of dissolution, and may yet be the subject of many additional operations. She still refuses to submit to efficient curative treatment, and often objects to such medication as may be necessary for her comfort. Indeed, she not unfrequently expresses a longing desire for death, that her sufferings may be ended.

*Lithotomy.—1 Case.*

Mr. O——, aged 19, rather a delicate youth, but not specially unhealthy, excepting perhaps for some seminal weakness, thoughtlessly introduced a button into the urethra, and allowed it to pass backwards into the bladder, two years before the operation for stone. Since this time, urinary troubles and irritable bladder have occasioned annoyance. Preparatory soundings revealed the presence of a foreign solid body in the bladder. The rectum and bladder having been properly attended to, he was subjected to lithotomy by the lateral operation, May 30, 1876. No great difficulty was encountered in making an entrance into the bladder, and the stone was withdrawn by aid of the scoop; chloroform was used.

The patient was left in a comfortable condition, a gum elastic catheter occupying the wound. The stone weighed 94 grains, and measured 1 inch by $\frac{3}{4}$ inch.

June 5th.—Sixth day after operation; hemorrhage to a moderate extent, from the bladder, was present yesterday, but ceased to-day; had a chill, followed by fever, the day before yesterday, with some vomiting; had chill yesterday; took quinine in sufficient quantity to affect the head; but little urine is escaping through the wound. Enemata, followed by a dose of salts, were administered yesterday, no action of the bowels having taken place since the operation ; a pretty free evacuation was secured ; quinine and injections to be continued, the latter, when needed, every second day.

June 11th.—Twelfth day ; for several days past quinine and cinchonidia have been discontinued, as being no longer indicated; indeed, no medicine has been administered, excepting saline laxatives, to empty the bowels, and morphia occasionally, to restrain excessive action. Four days ago, one or two drops of fecal matter appeared in the urine, escaping from the urethra, also a drop yesterday, as reported by the patient ; gas also escaped with the urine ; for the last 24 hours, no fecal matter nor gas has come away through the urinary canal, and no urine has escaped by the wound. Supposing a fistulous opening to exist betwixt the rectum and bladder, why, then, should fecal matter escape by the urethra, and not by the wound, while urine was still escaping by the wound ?

July 16th.—Forty-seventh day; the patient nearly well; wound almost healed up ; can retain his urine from 4 to 6 hours without inconvenience, discharging from 6 to 8 oz. at each micturition. No urine has passed by the wound for the last two weeks, and no further trouble is anticipated.

August 1st.—The recovery is complete.

In severe injuries, such as compound fractures of the extremities, the writer has frequently chosen a conservative practice, and although the injured limb may sometimes have been saved, it was in some instances left in a condition so defective, especially from damage to the soft parts, as to indicate that amputation would have been preferable. The limb was preserved, but the flesh remained unsound, and the slightest scratch or abrasion would result in an open wound. In two instances of this kind, now recollected, the patients would have preferred complete ablation of the damaged limb ; an artificial leg would have secured for them more comfortable locomotion than the imperfect though natural one.

*Imperforate Hymen.*—1 *Case.*

Maggie ——, 17 years of age, single, had never menstruated, though breasts and genitals were apparently well developed. A

gradual tumefaction of the abdomen had been going on for a long time, until now, May 6th, 1862, the enlargement simulates advanced pregnancy. She had recently suffered some tormina, tenesmus, and dysuria; to relieve these symptoms, she had taken anodynes and diuretics. Tenderness of the abdomen, especially in the hypogastric region, was now present. On May 14th, a vaginal examination was permitted, which revealed a fluctuating tumor in the vicinity of the hymen, and complete occlusion of the vagina at this point, the tumor measuring one by two inches, and extending backward from the meatus urinarius to the fourchette.

The parts named were painful and tender, and the obstructing membranes of no great thickness. Handling it, caused straining, as of a woman in labor. The catheter being introduced into the bladder, brought away no more than two or three ounces of urine, with no relief of the urgent symptoms, and without reducing the abdominal tumor. Imperforate vagina was diagnosticated, and an abcess lancet was passed through the membrane closing the vagina, which gave egress to three pints of menstrual blood. So strong was the expulsive pressure that a stream jetted out, extending two or three feet from the patient. The fluid was characteristic of the menstrual blood, but perhaps a little more sanious, and of more offensive odor. On the following day an obstruction to the further discharge was found to exist, which was broken down by the finger, and an amount of fluid equal to the first passed away, resulting in immediate relief of all the symptoms.

### *Hermaphrodism.*—1 *Case.*

The following case of uncertain sex, although not subject to the test of post mortem examination, is considered as sufficiently remarkable to justify being reported. A child of German parents was sent to me for inspection, by Dr. Thomas Runcie, an intelligent practitioner, in the country a few miles from Evansville, who had observed it from birth, and with doubts authorized its baptism as a boy. The child was nine weeks old, and apparently well nurtured; the scrotum was in appearance well developed, but no testes nor spermatic cords could be found. There was what seemed to be a penis of ordinary size, or an enlarged clitoris; its measurement was 1¼ inches in length and ½ inch in diameter, with perforate urethra, the perforation extending to the glans; the glans had its prepuce, only defective at the frenum, the whole organ being bound firmly down against the supposed scrotum. Just posterior to the glans, as this rested against the scrotum, was an opening in the perineum, into which a probe was introduced 1½ inches, as into a vagina. At first sight, therefore, the child seemed to be a boy, but closer inspection revealed the organ of the female, sufficiently at least to prove it of doubtful sex. To determine the sex beyond question, testes should have been found, on the one hand, or ovaries and uterus on the other. The child died soon after the inspection was made, but no dissection or post mortem examination was practicable.

*Occlusion of Os tincæ.*—1 *Case.*

Mrs. B., aged about 25 years, mother of two children, was seen Oct. 17th, 1875; she had been in labor six hours under the care of Prof. Davidson, the pains being strong and regular. The head of the child presented, and was pressed firmly against the anterior wall of the cervix, and was engaging in the excavation. Dr. Davidson had been entirely baffled in his efforts to find the os uteri. A few months back, Mrs. B. had been afflicted with ulceration of the os, for the relief of which caustic applications had been used, which seemed to offer an explanation of the unfortunate occlusion. Having been requested to assist in the case, the writer soon became convinced that no opening existed between the vagina and uterus; the cervix was low enough to be within reach, by the touch. At the posterior part of the tumor formed by the inferior portion of the uterus, a surface slightly uneven could be discerned, which was supposed to indicate the site of the occluded os. An artificial opening was resolved upon, and a crucial opening was made with a scalpel, through the intervening tissue, where it was supposed the natural opening ought to be, and happily the success of the operation was attested by the escape of a portion of amniotic fluid. The child was expelled without further difficulty, through the artificial os, about an hour afterward. Subsequently to the labor, the patient suffered from a slight attack of peritonitis, but finally made a good recovery. Subsequent examination proved that the newly-made os still remained open.

*Obstetrics.*

The observations made in the department of obstetrics, are predications upon the four positions, as laid down by Maggrin and Capuron, to wit: Two anterior and two posterior oblique positions of the presenting extremity of the child. I have notes of 822 consecutive cases of midwifery; of these 7 were twin cases, making 829 children. The presentations and positions were as follow, to wit:

| | | | |
|---|---|---|---|
| Vertex presentations, | | 793, or 97 | per cent. |
| Of these, | 1st position, | 431, or 54 | per cent. |
| | 2d position, | 308, or 31 | per cent. |
| | 3d position, | 13, or 1.6 | per cent. |
| | 4th position, | 10, or 1.2 | per cent. |

It is possible that some cases set down as 2d ought to be classed as 3d positions, the rotation of the head changing the occiput from the right sacro-iliac symphysis to the arch of the pubis, leading to misconception.

| | | | |
|---|---|---|---|
| Breech presentations, | | 18, or 2.1 | per cent. |
| to wit: | 1st position, | 11, or 61 | per cent. |
| | 2d position, | 4, or 22 | per cent. |
| | 3d position, | 3, or 16.6 | per cent. |
| Feet presentations, | | 2, or .2 | per cent. |
| to wit: | 2d position, | 1, or 50 | per cent. |
| | 3d position, | 1, or 50 | per cent. |
| Face presentations, | | 4, or .4 | per cent. |
| to wit: | 1st position, | 1, or 25 | per cent. |
| | 2d position, | 2, or 50 | per cent. |
| | 3d position, | 1, or 25 | per cent. |
| Trunk presentations, | | 3, or .3 | per cent. |
| to wit: | Side, | 2, or 67 | per cent. |
| | Chest, | 1, or 33 | per cent. |
| Presentations unknown, | | 10, or 1.2 | per cent. |

There were of male children 436, or 52.5 per cent.
of female children 393, or 47.4 per cent.

The results in the 822 labors, for one month, were: Mothers died, 8, or 1 per cent., to wit: From flooding, 3; other causes, 5. Children still-born, 28, or 3.3 per cent., to wit: From hydrocephalus, 1; general dropsy, 1; prematurity, 7; other causes, 13.

Of the 14 twins there were:

Presentation of vertex, 9, or 66 per cent.
" of breech, 3, or 20 per cent.
" of feet, 2, or 14 per cent.

Of obstetrical operations in the 822 cases, there were 21, or 2.5 per cent., as follow: Forceps cases, 10, or 1.2 per cent. of the whole number. Of these cases, children lost, 2, or 20 per cent.; women lost, 0.

Version cases, 11, or 1.3 per cent. of the whole. Of these cases, children lost, 4, or 36 per cent.; women lost, 0.

Of the obstetrical operations herein reported, it may be observed that but few of them belong properly to my own practice, being consultation cases. I have kept them separately, so that from my own list the proportion of operations to cases may be more fairly represented. According to this statement, no more than 2½ per cent. of cases required operative aid for completing the delivery. It may, however, possibly be true, that more mothers, as well as children, might have been saved had more operations been resorted to. Of late years I do not delay operation to so late a period of the labor as formerly.

The secale cornutum has seldom been used by the writer with a view to expedite labor, for the last 25 years, after a pretty extensive trial in early professional life; it is still administered, however, to promote contractions after delivery, and thus to prevent or check, post partum hemorrhage. The following are considered valid objections to its use as an oxytocic: 1st. It frequently fails to effect the expulsion of the child, in which case the woman is in a worse condition than if it had not been used, both from general exhaustion and special exhaustion of uterine power. 2d. The child, when expelled by the aid of the ergot, is liable to be dead, or dangerously asphyxiated, probably from obstruction of circulation through the cord, from continuous ergotic contractions. 3d. In all cases adapted to the use of ergot, turning or forceps will be a better resource, being more expeditious, as well as safe, for both mother and child; and these operations are made more difficult after the ergot has been unsuccessfully used.

*Inversion of Uterus.*—1 *Case.*

Mrs. C——, aged about 22, in labor with third child, July 2, 1864, had inversion of uterus after first, and partial inversion after second labor. Immediately after the escape of the placenta, the fundus uteri gradually descended, occupying first the uterine, and next the vaginal canal, until finally, the whole organ had passed the os. Its restoration having been undertaken at once, was effected by gently indenting its most dependent point with the fingers, and gradually pushing it upward, until at length it was returned to its normal position. This effected, its full contraction was secured by mechanical irritation applied both externally and internally, the patient being carefully watched, until all danger from relaxation had passed over. From the strong tendency to inversion in this, as well as the two preceding labors, it would seem that some peculiarity of organization of the woman existed, favoring the mal-position, at least no other cause was perceptible, excepting a natural feebleness, and relaxed condition of the system generally.

*Version.*—27 *Cases.*

In my note book I find 41 cases of turning. The following were the conditions making the operation necessary, to wit.:

Placenta prævia, 6, or 14 per cent., only one being primipara.

Inertia, 13, or 30 per cent.

Descent of hand, 7, or 16 per cent.

Descent of hand and funis, 4, or 9.5 per cent.

Contracted pelvis, 4, or 10 per cent., one of these being primipara.

Flooding, 3, or 7.3 per cent.

Face presentation, 1, or 2.3 per cent.

Shoulder presentation, 2, or 4.7 per cent.

The turning was in every case podalic, and effected by seizing and drawing down the first foot found.

In the 6 placenta prævia cases, the membranes were unbroken at the beginning of the operation; in the remaining 35 the membranes had been previously ruptured in all excepting 2 cases, as follows, to wit.:

Ruptured 1 hour or less, in 8 cases.
" from 1 to 2 hours, in 2 cases.
" from 2 to 5 hours, in 16 cases.
" from 5 to 10 hours, in 5 cases.
" two days, in 1 case.
" three days, in 1 case.

Most of these cases were not seen until they had been for a longer or shorter time under the care of others, most of them female midwives, consequently an earlier operation was impracticable. That some of the cases resulted favorably, under the disadvantage of having had the waters long drained off, gives encouragement not to abandon such cases as not suitable for turning, but, on the contrary, in many instances, they may be successfully managed, even to a greater extent than the statements of some authors would lead us to believe. In these cases the mother's life may generally be preserved, and if the child be still vigorous, its life also, in a reasonable number of cases, may be saved.

The following are some of the most interesting of these cases:

*Placenta Prævia.*—6 *Cases.*

1. Mrs. R——, aged about 40, mother of several children, called in Prof. Bray at 9 o'clock a. m., Feb. 4th, 1853. The patient had been suffering more or less for two or three months, from uterine hemorrhage, which, until now, had not been considered alarming. The writer was called at 9 p. m. The patient had been flooding pretty freely, and had suffered moderate labor pains. The flooding flow was increased by each uterine contraction. Prof. Bray had already diagnosticated placenta prævia, which was acquiesced in after a vaginal examination. The os uteri was open to the size of a silver dollar—rather rigid; the vagina filled by a large coagulum. The placenta was attached to the left side of the cervix, extending entirely across the orifice, the edge being quite perceptible on the right side. By this time, the patient was becoming much weakened from loss of blood. Delivery by turning was decided upon, as being indicated, without delay. The hand, on being introduced into the amniotic sac, revealed a left anterior presentation of the vertex; the left foot was secured and brought down, and the delivery completed in about thirty minutes. Some delay was caused by the head lingering for a short time after the body was born, to the great detriment of the infant; it was apparently at term, and asphyxiated; pulsation of the heart and large arteries continued for an hour, but natural respiration could not be established. The woman was much exhausted, but finally made a good recovery.

2. Mrs. ——, a German woman—43 years of age, had been in care of a midwife, since 9 o'clock a. m., July 5th, 1853. Prof. Byford saw her about 4 p. m. The midwife had noticed however, that the afterbirth was coming first, which view was confirmed by the touch by Prof. Byford. The writer was now called in, and finding the os uteri dilated to the size of a silver dollar, relaxed and dilatable, the patient in the beginning of the ninth month of gestation, with considerable flooding, the placenta extending half across the os, immediate delivery by turning was agreed upon. The hand was made to enter the uterus without difficulty, the membranes ruptured on the right side, this being opposite to that occupied by the placenta, the child's right foot was seized and brought down, and the delivery completed, by the second position of the breech. The child soon commenced crying, the pleasantest music to the parturient woman. The infant was rather feeble, from being premature; the woman was left in good condition, with the uterus well contracted.

3. Mrs. ——, a German woman about 45 years of age, supposed to be 8½ months pregnant, was attacked with flooding and labor pains, in August, 1854. She was at first under the care of an ignorant midwife for four days, at which time Dr. Weever, an intelligent physician, was called to see her. The patient having lost a large quantity of blood, was greatly exhausted. The writer was asked to see her by Dr. Weever, at which time the following condition was present: Patient apparently almost bloodless and *in articulo mortis*, pulse not perceptible at the wrist, extremities cold, uterine contractions feeble, but still continuing, flow of blood still copious. Examination per vaginam, disclosed the presence of a granular fibrous mass within the os uteri, which was decided to be the placenta attached to the cervix ; on each return of pain, this body was retracted to the right side of the os, the membranes being noticeable at the left side. Immediate delivery by version was resolved upon ; Dr. Weever, however, expressing the opinion that the patient could not survive an hour, and might expire before the operation could be completed. The hand being introduced along the left side of the cervix, toward the presenting membranes, the attachment of the placenta being disturbed as little as possible, it was then forced through the membranes, when the feet were secured and brought down and the child delivered. The placenta came away without difficulty. The period that elapsed from the introduction of the hand to the delivery of the child, did not exceed ten minutes. The child had been dead for a considerable time, as shown by the slipping of the skin, and other signs of decomposition. The woman did not rally, and died two days after delivery.

4. Mrs. S——, aged 26 years, has generally enjoyed good health, mother of two children, has been troubled by frequent floodings for the last two months. On examination per vaginam, on September 9, 1863, the placenta was found attached to the right side of the cervix, extending entirely across the os uteri; the patient had lost much blood during the last 24 hours ; ergot was administered yesterday, followed by slight labor pains; each pain, and also every effort to cough, occasioned a fresh gush of blood. Thc child was delivered by podalic version this morning at 9 o'clock ; the child was still-born; the mother died from exhaustion approaching to syncope, two hours after delivery. The delivery was effected without difficulty, and during and subsequent to the operation, but little blood was lost.

5. Mrs. McK——, aged 37 years, mother of several children, of delicate health, was seen April 12th, 1865. She had flooded moderately several days ago, which was relieved by remaining quiet; last evening it returned with considerable violence, and has continued more or less actively to-day. There was little pain until 2 p. m., April 13; at this time she was found without pulse at the wrist, with cold extremities, and every indication of sinking. Examination by the vagina revealed that the os uteri was dilated to the diameter of an inch, and dilatable; the placenta was implanted over the os uteri. The hand was passed through the cervix after overcoming considerable resistance, the child's feet seized, and the delivery accomplished. It was apparently a 6½ month child, and after gasping for a short period, it expired. The woman's prostration lasted for some time, but was attended with little subsequent hemorrhage. Reaction was secured by quietness, and a moderate use of stimulants, and in the end the patient made a good recovery.

6. Mrs. G——, aged about 30, mother of three children, two of them being twins; her last labor occurred about three years since. The services of the writer were requested, May 26, 1872. She had been under the care of Dr. Thompson for about a month, for repeated floodings. Dr. Thompson diagnosticated placenta prævia, and had applied all the ordinary means for arresting the hemorrhage; quietness and the tampon had been diligently applied. On examination, the placenta was found to be situated over the mouth of the uterus, chiefly on the right side of the cervix; the membranes being easily recognized at the left of the os; the orifice was open to the size of a silver dollar, and soft; the patient was considerably exhausted and somewhat feverish. Immediate delivery by turning was deemed advisable. By using a moderate amount of force, the hand was passed through the cervix, more force, indeed, was used, than would have been advisable in a less urgent case; the os gradually relaxed, however, and admitted the hand into the cavity of the uterus; the right foot was seized and brought down; the head being in the pelvis a short time after the body came away, but was finally delivered, and after a few minutes the child commenced breathing. The woman suffered for several days, from shock and loss of blood, but rallied well. From her own preference chloroform was not used; the child weighed 5½ pounds; finally, both mother and child did well.

*Descent of Hand.—2 Cases.*

7. Mrs. ——, aged 24 years, mother of 4 children, of good constitution, was taken in labor on the morning of August 4,

1854. A German midwife had charge of the case until 1 p. m., when the membranes ruptured, and the child's hand descended into the vagina. The writer was then requested to see the patient, the water having passed off half an hour before. Delivery by turning was decided upon, the hand was introduced into the uterus, the right foot secured and brought down, and the delivery completed in twenty minutes; the child was asphyxiated, but recovered in twenty minutes, and both mother and child did well.

8. In this case, a German woman, name unknown, of middle age, mother of several children, had placed herself under a midwife at the commencement of labor. When the membranes gave way, the child's left hand descended through the vagina, and appeared externally. Dr. John T. Walker was called in to render assistance, and sent for the writer. The hand was discovered external to the vulva, face anterior, and head in left iliac fossa. The water had now been drawn off more than an hour. After passing the hand into the uterine cavity, the right knee was grasped and brought down, the protruding hand returning spontaneously into the womb, as the feet descended. A male child was safely delivered, which commenced crying in a few minutes, and breathed well. Both mother and child did well.

[TO BE CONTINUED.]

